# Adipose tissue‐derived small extracellular vesicles and blood–brain barrier function in adults with overweight and obesity

**DOI:** 10.1113/EP092878

**Published:** 2026-01-28

**Authors:** Shalini Mishra, Yixin Su, Sangeeta Singh, Ashish Kumar, Fang‐Chi Hsu, Gagan Deep, Tina E. Brinkley

**Affiliations:** ^1^ Department of Internal Medicine, Section on Gerontology and Geriatric Medicine Wake Forest University School of Medicine Winston‐Salem North Carolina USA; ^2^ Sticht Center for Healthy Aging and Alzheimer's Prevention Wake Forest University School of Medicine Winston‐Salem North Carolina USA; ^3^ Division of Public Health Sciences, Department of Biostatistics and Data Science Wake Forest University School of Medicine Winston‐Salem North Carolina USA; ^4^ Atrium Health Wake Forest Baptist Comprehensive Cancer Center Wake Forest University School of Medicine Winston‐Salem North Carolina USA

**Keywords:** adipose tissue, obesity, small extracellular vesicles, weight loss

## Abstract

Obesity is associated with adverse changes in brain structure and function, in part, through crosstalk between adipose tissue (AT) and the brain. AT releases small extracellular vesicles (sEV) that can cross the blood–brain barrier (BBB) and modulate multiple pathophysiological pathways, including BBB function; however, this has never been investigated. We characterized circulating adipose tissue‐derived sEV (sEV^AT^) in adults with overweight and obesity and examined their effects on the BBB. The impact of adiposity and weight loss on these outcomes was also examined. sEV^AT^ were isolated from the plasma of 29 adults (79% male; 93% White; mean age 66.2 ± 7.0 years; mean body mass index 36.0 ± 6.8 kg/m^2^) randomized to cardiac rehabilitation (CR) alone or CR plus a behavioural weight loss intervention (CR+WL). Following characterization of sEV^AT^ size, concentration and total protein content, we assessed their effect on BBB permeability using an in vitro model. hCMEC/D3 cells were treated with sEV^AT^, and transendothelial electrical resistance (TEER) was measured at 0, 24, 48 and 72 h. Our findings show that sEV^AT^ treatment decreased TEER by 40%, with a significantly lower TEER at 72 h compared with controls (23.138 ± 1.209 vs. 28.724 ± 1.613 Ω cm^2^, *p *= 0.012). TEER was also lower in participants with higher body mass index and body fat. However, we found no difference in TEER between the CR and CR+WL groups and no significant intervention effects on sEV^AT^ characteristics or TEER. In conclusion, higher plasma sEV^AT^ concentrations in adults with overweight and obesity are associated with greater adiposity, which might contribute to reductions in BBB function.

## Abstract (highlights)

- **What is the central question of this study?**
  Small extracellular vesicles secreted from adipose tissue (sEV^AT^) might impair blood–brain barrier function. We developed an in vitro model to assess the effect of sEV^AT^ on the blood–brain barrier in adults with overweight and obesity randomized to one of two lifestyle interventions.
- **What is the main finding and its importance?**
  Treatment of hCMEC/D3 cells with sEV^AT^ decreased transendothelial resistance in both groups, with no significant intervention‐related effects. Higher plasma sEV^AT^ concentrations were associated with greater adiposity, suggesting that sEV^AT^ might contribute to the adverse effects of obesity on brain health.

## 1 INTRODUCTION

Overweight and obesity are associated with adverse changes in brain structure and function, independent of related comorbidities, including hypertension, diabetes and cardiovascular disease (Gustafson et al., 2004a, 2004b; 2007; Pannacciulli et al., 2006; Ward et al., 2005). Multiple lines of evidence also indicate that brain microvascular endothelial cells and the blood–brain barrier (BBB), which tightly regulate the selective transport of nutrients and hormones across the CNS and protect the brain from toxic substances in the circulation, might also be impaired in individuals with overweight and obesity (Avolio et al., 2018; Rhea et al., 2017). This dysfunction often occurs in conjunction with altered cellular energy metabolism and peptide transport, increased infiltration of immune cells and downregulation of tight junction proteins (Gustafson et al., 2007; Rhea et al., 2017). Because cerebral vessels are not surrounded by adipose tissue (AT), the effects of obesity on the cerebral vasculature are most likely related to changes in blood pressure, innervation and circulating factors. Notably, AT secretes a number of mediators (collectively known as adipokines), such as adiponectin and leptin, that act as endocrine factors to modulate brain metabolism, neuroinflammation and neurodegeneration (Hallschmid et al., 2009; Kloting et al., 2011; Kos et al., 2007; Schmid et al., 2016; Zhao et al., 2011). Although such pathological changes are likely to be driven by excess adiposity and related metabolic consequences, very little is known about how AT promotes BBB dysfunction.

Small extracellular vesicles (sEV) are lipid membrane‐bound nanovesicles (size <200 nm), which are secreted extracellularly by every cell in the body. sEV contain a diverse cargo of lipids, proteins, metabolites and nucleotides, which participate in intercellular communication, removal of unwanted proteins and transfer of pathological biomolecules between cells (Bellingham et al., 2012; Colombo et al., 2014; Valadi et al., 2007). sEV secreted from AT (sEV^AT^) play a role in interorgan communication and might alter the function of recipient cells (Isaac et al., 2021; Kita et al., 2019; Koeck et al., 2014). Studies show that sEV^AT^ are capable of inducing insulin resistance, inflammation and fibrosis in target organs, particularly in the setting of adipocyte hypertrophy and obesity (Deng et al., 2009; Ferrante et al., 2015). In addition, the circulating levels and microRNA content of sEV^AT^ are altered in obese subjects (Eguchi et al., 2016; Hubal et al., 2017; Witczak et al., 2018). Although the ability of sEV^AT^ to mediate communication between AT and the brain remains largely unknown, recent data indicate that sEV^AT^ are able to cross the BBB (Alvarez‐Erviti et al., 2011) and could be taken up by brain cells, where they affect signalling pathways that regulate energy homeostasis (Gao et al., 2019) and ultimately modulate cognitive function (Wang et al., 2022).

Weight loss has well‐known benefits for the cardiovascular system, and these beneficial effects are likely to extend to the cerebral vasculature. There is some evidence that weight loss might improve brain endothelial function and repair BBB function in individuals with obesity (Rhea et al., 2017). In addition, the high circulating levels and altered microRNA content of sEV^AT^ observed in obesity are improved with weight loss and are correlated with improvements in glycaemic control (Eguchi et al., 2016; Hubal et al., 2017; Witczak et al., 2018). However, whether weight loss‐induced changes in sEV^AT^ are correlated with improvements in cerebrovascular health remains unknown. To explore the hypothesis that sEV^AT^ alter BBB function and that this effect can be modulated by excess adiposity and diet‐induced weight loss, we leveraged stored plasma samples from middle‐aged and older adults enrolled in a weight loss intervention study.

## 2 MATERIALS AND METHODS

### 2.1 Ethical approval

This study was approved by the Wake Forest University School of Medicine Institutional Review Board (IRB00046110) and was registered at clinicaltrials.gov (NCT03423238). All study procedures were conducted in accordance with the *Declaration of Helsinki*, and all participants provided written informed consent prior to data collection.

### 2.2 Study population

The present analysis includes 29 adults aged ≥40 years with coronary heart disease and a body mass index (BMI) of ≥25 kg/m^2^ who were enrolled in a randomized clinical trial designed to test the feasibility of adding a behavioural weight loss intervention during cardiac rehabilitation. All participants were cognitively normal based on a Montreal Cognitive Assessment (MoCA) score of ≥22 (Islam et al., 2023). The full inclusion/exclusion criteria for this study have been described previously (Brinkley et al., 2023). Participants were randomized to cardiac rehabilitation alone (CR) or cardiac rehabilitation plus a behavioural weight loss programme (CR+WL) for 6 months.

### 2.3 Interventions

All 29 participants were enrolled in an exercise‐based CR programme, consisting of three exercise sessions and one health education class per week for 6 months. Participants randomized to the CR+WL group (*n* = 18) also attended weekly/biweekly individual behavioural counselling sessions with a registered dietitian and were provided with meal replacements and a personalized meal plan designed to achieve a target weight loss of ≥5%. Details of the CR programme and behavioural weight loss intervention have been described previously (Brinkley et al., 2023).

### 2.4 Body weight, adiposity and cardiometabolic risk factors

Anthropometric measurements, including height, weight, waist and hip circumference and waist‐to‐hip ratio, were determined using standard procedures. Dual‐energy X‐ray absorptiometry was performed on a Horizon scanner (Hologic Inc., Belford, MA, USA) using the BodyLogic scan and APEX analysis software (v.5.5.3.1) to assess whole‐body and regional measures of fat and lean tissue, in addition to visceral and subcutaneous adipose tissues (VAT and SAT, respectively) (Brinkley et al., 2023). Blood pressure and fasting glucose, insulin, haemoglobin A1c and lipid levels were measured using standard procedures. Insulin resistance was assessed using the homeostasis model assessment (HOMA‐IR) (Matthews et al., 1985).

### 2.5 Isolation of sEV^AT^

**FIGURE 1:**
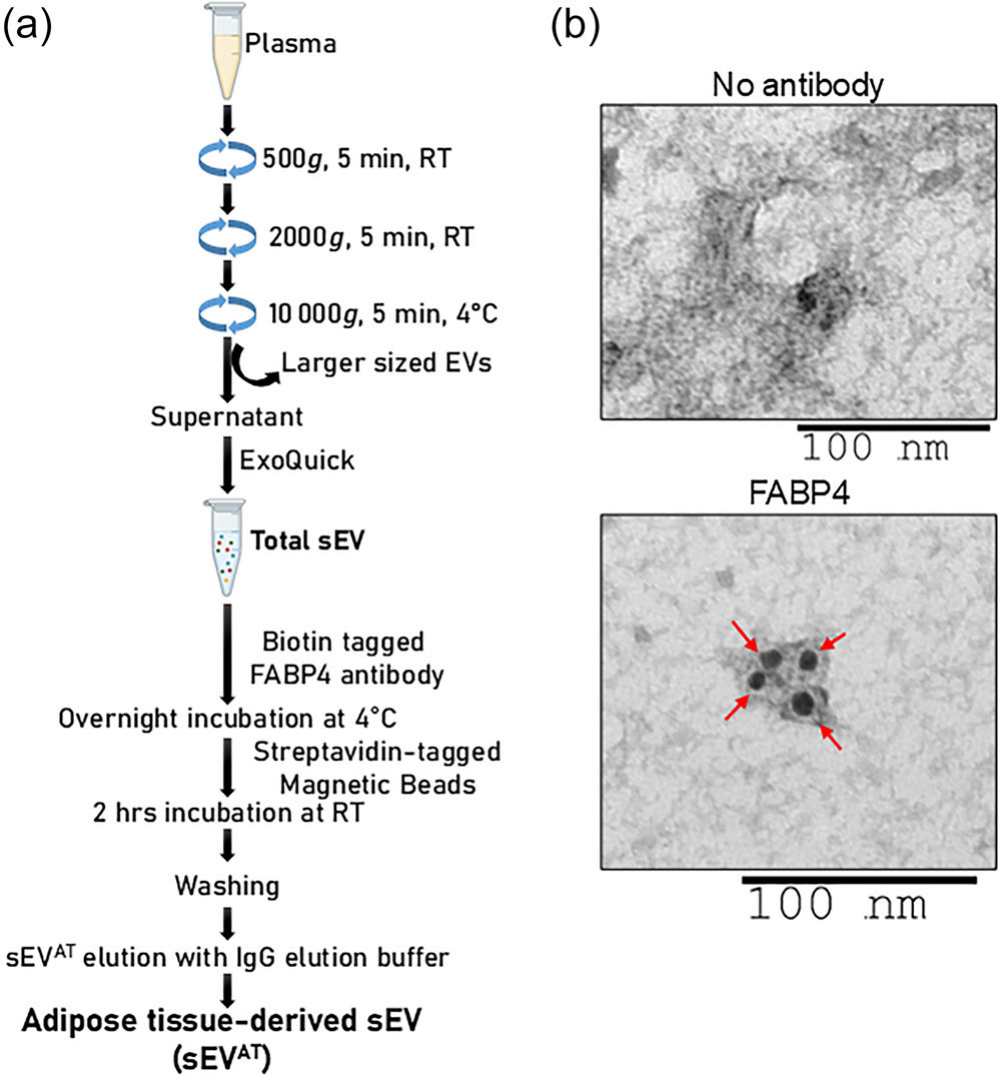
**Isolation and characterization of sEV^AT^ from plasma of participants randomized to CR and CR+WL. (a)** Diagrammatic presentation of steps involved in sEVAT isolation from plasma. **(b)** Representative images captured at 98,000X magnification showing the size and surface expression of FABP4 (indicated by red arrows) by immunogold labeling and TEM (scale bar: 100 nm) (n=5).

Fasting blood samples were collected via venipuncture and processed immediately for storage at −70°C. Total sEV and sEV^AT^ were isolated from plasma using a combination of precipitation and immuno‐pull down methods, as shown in Figure 1a. Briefly, plasma samples (volume made up to 1 mL with PBS) were differentially centrifuged at 500, 2000 and 10 000*g* to remove debris and large‐sized extracellular vesicles. The supernatant was then incubated with 300 µL thromboplastin D for 1 h at room temperature (RT), mixed with 700 µL PBS containing proteases and phosphatase inhibitor, and centrifuged at 1500*g* for 20 min. The supernatant was mixed with 504 µL of ExoQuick (EXOQ; System Biosciences, Inc., Mountainview, CA, USA) and incubated for 1 h at 4°C. Samples were then centrifuged at 1500*g* for 30 min at 4°C. The resulting pellet was resuspended in 1 mL of PBS, and protein concentration (in micrograms per microlitre) was measured using NanoDrop.

To isolate sEV^AT^, 1500 µg of total sEV was incubated with 6 µg of biotin‐tagged fatty acid binding protein 4 (FABP4) antibody (ARP33794_P050‐Biotin, Avivasysbio) overnight at 4°C with constant mixing. After antibody incubation, streptavidin‐tagged magnetic beads were added to the samples and incubated for 2 h at RT with continuous mixing. Thereafter, beads were washed with TBST (TBS with 0.1% Tween‐20) twice, and 200 µL of IgG elution buffer (21004; ThermoScientific) was added to the beads for 10 min to elute sEV^AT^. Beads were magnetized, and the supernatant was collected in tubes containing 20 µL of 1 M Tris buffer (pH 9) to collect sEV^AT^ (Hubal et al., 2017; Mishra et al., 2025). The protein concentration of sEV^AT^ was measured using the Bicinchoninic Acid (BCA) method according to the vendor's protocol (Pierce BCA Protein Assay Kits; 23225; Thermo Fischer Scientific).

### 2.6 Characterization of sEV^AT^

The sEV^AT^ were characterized by nanoparticle tracking analysis (NTA) using a NanoSight NS300 system (Malvern Instruments Ltd, UK). The instrument was primed with PBS (pH 7.4), maintaining the temperature at 25°C. Five videos (30 s per video) were captured for each sample using NTA software (version NTA 3.4) to assess the concentration and size distribution of the particles (Kumar et al., 2021, 2023; Pait et al., 2024). Polystyrene beads measuring 100 and 200 nm (Malvern Instruments Ltd, UK) were run as standards.

### 2.7 Immunogold labelling and transmission electron microscopy

Immunogold labelling was performed to confirm FABP4 expression on the sEV^AT^ surface using a previously published protocol (Mishra et al., 2023). Copper grids (with carbon‐coated formvar film) were activated by incubation with 100% ethanol for 20 min. Next, 30 µL of sEV^AT^ were fixed by adding an equal amount of 4% paraformaldehyde for 10 min at RT. Fixed sEV^AT^ were incubated with grids for 1 h at RT. The grids were then washed three times by putting them in PBS containing 50 mM glycine for 5 min. Grids were incubated with blocking buffer (0.5% bovine serum albumin in PBST) for 30 min at RT, then incubated with FABP4 antibody (1:100) overnight at 4°C. After washing with PBST (3 × 5 min), grids were incubated with secondary antibody (anti‐rabbit gold IgG; 1:100) for 2 h at RT in the dark. After washing with PBST (3 × 5 min), grids were incubated with 2.5% glutaraldehyde for 5 min and washed with PBS (7 × 5 min). Grids were incubated with 1% uranyl acetate for 1 min, then with distilled water for 2 min. Images of grids were captured using transmission electron microscopy (FEI Tecnai Spirit transmission electron microscope system) at ×98 000 magnification.

### 2.8 Brain endothelial permeability assay

**FIGURE 2:**
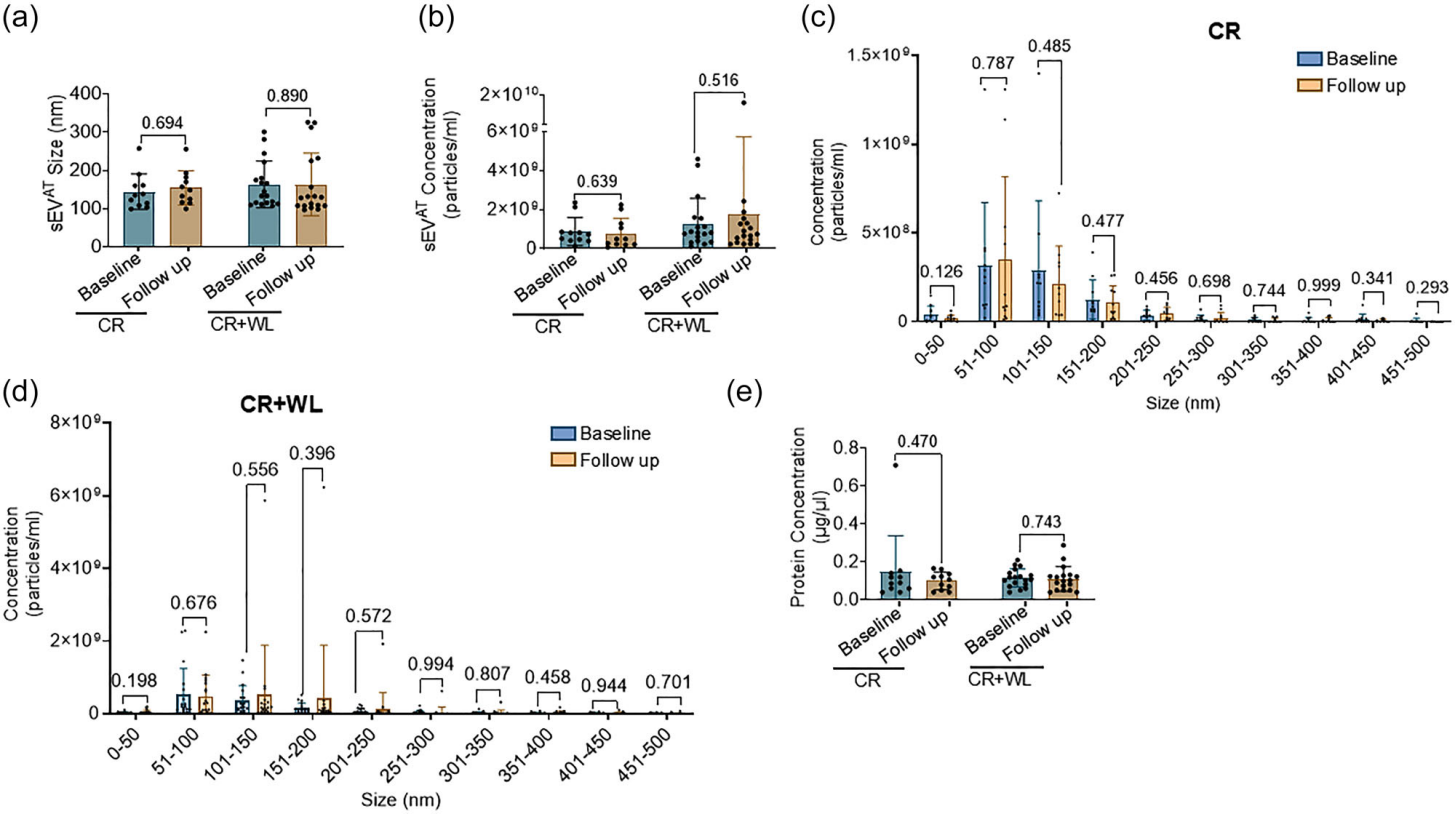
**Schematic representation of protocol for *in vitro* BBB experiment**. Transendothelial electrical resistance was recorded following seeding of human brain cerebral microvessel endothelial cells in Transwell inserts and treatment with sEV^AT^.

The brain endothelial permeability assay was performed using an in vitro model for BBB studies (Niego & Medcalf, 2013; Niego et al., 2012, 2017). This in vitro assay was performed using samples from 19 of the 29 participants. An outline of the experiment is shown in Figure 2. Briefly, 25 000 human brain cerebral microvessel endothelial cells (hCMEC/d3) (SCC066, Sigma) were seeded in the apical chamber of 0.33 cm^2^ Transwell inserts (3 µm pore size, Corning, NY, USA) coated with rat tail collagen type I (5 µg/cm^2^). Cell medium was changed every 48 h. The transendothelial electrical resistance (TEER) was recorded using the EVOM™ Epithelial Volt/Ohm Meter 3 (EVOM3) connected to a STX2‐PLUS electrode (World Precision Instruments, Sarasota, FL, USA). TEER recordings started at day 7 of cell seeding to ensure proper establishment of cell monolayers and formation of the endothelial barrier. Once the TEER values stabilized (at day 11), the medium was changed to exo‐free medium, and cells were treated either with vehicle control (PBS) or 10 µg of sEV^AT^ isolated from blood samples collected at the baseline and 6 month follow‐up visits. TEER was measured after 24, 48 and 72 h of sEV^AT^ treatment along with controls. Final TEER values were calculated as: (Measured TEER value − blank TEER value) × area of insert. Higher TEER values (in ohms per centimetre squared) reflect better BBB function (i.e., lower permeability).

### 2.9 Statistical analyses

The two‐sample *t*‐test was used to compare intervention effects on body fat, sEV^AT^ characteristics and TEER. A paired *t*‐test was used to assess within‐group (i.e., CR and CR+WL) changes from baseline to follow‐up. Repeated‐measures analysis with the unstructured covariance matrix was used to examine the effects of sEV^AT^ treatment on TEER over time. Baseline TEER, group (sEV^AT^‐treated cells vs. controls or CR vs. CR+WL), time, and group by time interaction were included in the model. Contrasts were used to compare group means at each time point. Spearman's rank correlation coefficients were used to examine associations between adiposity, sEV^AT^ and TEER outcomes. Due to the nature of this pilot study, multiple comparisons were not considered. For all analyses, statistical significance was set at *p* ≤ 0.05. All analyses were performed using SAS v.9.4 (SAS Inc., Cary, NC, USA).

## 3 RESULTS

### 3.1 Baseline characteristics

The baseline demographic, clinical and sEV^AT^ characteristics of the study population are shown in Table 1. Overall, the mean age of the participants was 66.2 ± 7.0 years, 93% were White, and 21% were female. In addition, 79% were obese, with an average BMI of 36.0 ± 6.8 kg/m^2^ and an average total body fat of 40.6% ± 5.4%. Abdominal and visceral fat were also high, with an average waist circumference of 115.3 ± 15.0 cm, abdominal VAT area of 235.8 ± 67.7 cm^2^ and VAT/SAT ratio of 0.52 ± 0.18. In general, systolic blood pressure, fasting glucose and haemoglobin A1c levels were in the elevated range, whereas lipid levels were well controlled. Characterization of sEV^AT^ by immunogold labelling and transmission electron microscopy confirmed the surface expression of FABP4 (Figure 1b), and NTA data showed that the average size and concentration of the sEV^AT^ were 156.03 ± 55.92 nm and 1.12 ± 1.12 × 10^9^ particles/mL, respectively (Table 1). In addition, the average total protein content of sEV^AT^ was 0.127 ± 0.121 µg/µL, as estimated by the BCA method (Table 1). All baseline characteristics were similar between intervention groups.

**TABLE 1:** Baseline characteristics of study population overall and stratified by intervention group.

Participant characteristics	Overall (*n* = 29)	CR+WL (*n* = 18)	CR (*n* = 11)
Demographics			
Age, years	66.2 (7.0)	66.0 (8.1)	66.3 (5.4)
Female sex	6 (20.7)	3 (16.7)	3 (27.3)
White race	27 (93.1)	17 (94.4)	10 (90.9)
MoCA score	25.8 (1.8)	25.8 (1.6)	25.8 (2.1)
Adiposity measures			
BMI, kg/m^2^	36.0 (6.8)	35.4 (6.5)	37.0 (7.6)
Waist circumference, cm	115.3 (15.0)	115.1 (15.4)	115.6 (15.1)
Waist‐to‐hip ratio	0.98 (0.09)	1.00 (0.10)	0.95 (0.08)
Total fat mass, kg	44.1 (13.9)	42.8 (14.2)	46.1 (13.6)
Total body fat, %	40.6 (5.4)	40.3 (5.4)	41.0 (5.7)
Android fat, %	45.1 (5.4)	45.4 (5.3)	44.7 (5.7)
Gynoid fat, %	39.8 (5.7)	39.5 (6.2)	40.4 (5.0)
Abdominal VAT, cm^2^	235.8 (67.7)	246.5 (78.9)	218.1 (41.1)
Abdominal SAT, cm^2^	476.0 (142.3)	464.9 (130.0)	494.2 (165.5)
VAT/SAT ratio	0.52 (0.18)	0.55 (0.20)	0.47 (0.11)
Cardiometabolic risk factors			
Systolic BP, mmHg	132.2 (20.2)	130.7 (18.2)	134.8 (23.9)
Diastolic BP, mmHg	72.5 (12.2)	71.2 (11.2)	74.8 (14.0)
Total cholesterol, mg/dL	121.3 (25.0)	118.7 (28.0)	125.7 (19.6)
LDL cholesterol, mg/dL	57.1 (23.5)	54.2 (26.8)	61.8 (17.1)
HDL cholesterol, mg/dL	40.6 (8.4)	40.2 (10.1)	41.2 (4.8)
Triglycerides, mg/dL	118.2 (42.0)	121.3 (48.1)	113.1 (30.9)
Haemoglobin A1c, %	6.0 (0.9)	5.8 (0.7)	6.4 (0.9)
Fasting insulin, mg/dL	21.8 (13.8)	19.9 (11.2)	24.9 (17.5)
Fasting glucose, mg/dL	110.8 (41.8)	101.8 (39.6)	125.5 (42.7)
HOMA‐IR	6.1 (4.3)	5.1 (3.2)	7.7 (5.4)
sEV^AT^ characteristics			
Size, nm	156.03 ± 55.93	163.14 ± 61.02	144.41 ± 46.77
Concentration, ×10^9^ particles/mL	1.12 ± 1.12	1.27 ± 1.30	0.87 ± 0.72
Protein content, µg/µL	0.127 ± 0.121	0.114 ± 0.048	0.147 ± 0.191

*Note*: Table values are the mean (SD) or *n* (%). Abbreviations: BP, blood pressure; BMI, body mass index; CR, cardiac rehabilitation; CR+WL, cardiac rehabilitation plus a behavioural weight loss intervention; HDL, high‐density lipoprotein; HOMA‐IR, homeostasis model assessment of insulin resistance; LDL, low‐density lipoprotein; MoCA, Montreal Cognitive Assessment; SAT, subcutaneous adipose tissue; sEV^AT^, adipose tissue‐derived small extracellular vesicles; VAT, visceral adipose tissue.

### 3.2 Intervention effects

**FIGURE 3:**
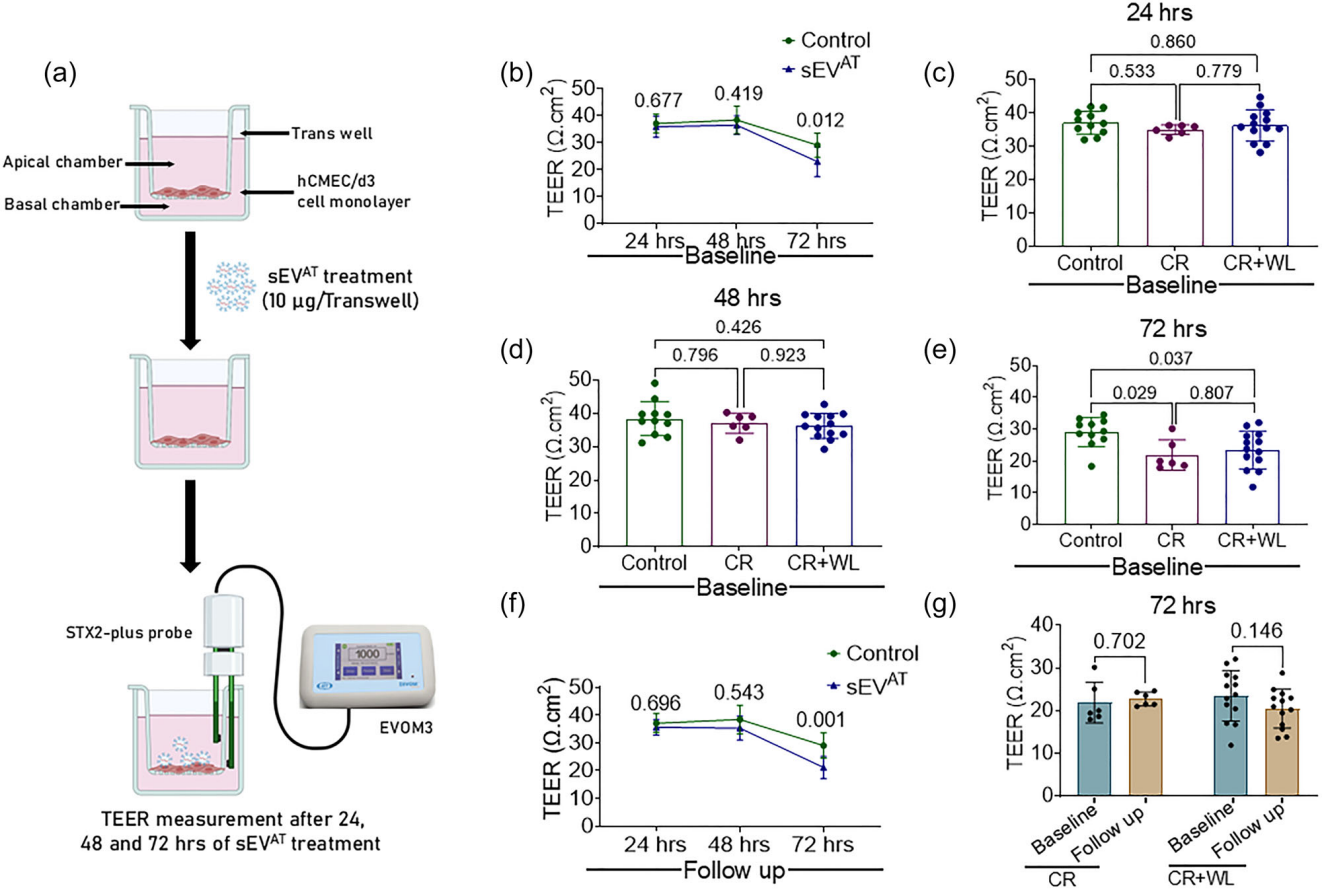
**Intervention effects on the size, concentration, and protein content of sEV^AT^**. Bar graphs representing the mean size **(a)** and concentration **(b)** of sEV^AT^ in the CR and CR+WL groups at baseline and 6‐month follow up. Bar graph representing the concentration of sEV^AT^ at different size ranges in the CR **(c)** and CR+WL **(d)** groups at baseline and follow‐up. **(e)** Bar graph representing the total protein content of sEV^AT^ at baseline and follow‐up in the CR and CR+WL groups. Bar graph data are shown as mean ± SD (CR: n=11; CR+WL: n=18).

Consistent with the main study results, in the present analysis, participants who were randomized to the CR+WL group had significantly greater weight loss and fat loss and greater improvements in body fat distribution compared with those in the CR group (Table 2). Changes in other cardiometabolic risk factors were similar between groups. The sEV^AT^ characteristics, including average size (Figure 3a), concentration (Figure 3b), size distribution (Figure 3c and 3d) and the average protein content (Figure 3e), were similar at baseline and follow‐up in both the CR and CR+WL groups. There were no statistically significant intervention‐related changes in sEV^AT^ size (CR+WL, 2.08 ± 62.91 nm vs. CR, 6.44 ± 52.63 nm, *p *= 0.849), concentration (CR+WL, 0.51 ± 3.25 × 10^9^ particles/mL vs. CR, −0.09 ± 0.64 × 10^9^ particles/mL, *p *= 0.552) or total protein content (CR+WL, −0.005 ± 0.064 µg/µL vs. CR, −0.047 ± 0.207 µg/µL, *p *= 0.428) in either group.

**TABLE 2:** Intervention effects on body weight, adiposity and cardiometabolic risk factors.

Participant characteristics	Overall (*n* = 29)	CR+WL (*n* = 18)	CR (*n* = 11)	*p*‐Value
ΔBody weight, kg	−4.9 (5.9)	−7.0 (6.4)^*^	−1.7 (3.3)	**0.0170**
ΔBMI, kg/m^2^	−1.7 (1.9)	−2.3 (2.0)	−0.6 (1.2)	**0.0165**
ΔWaist circumference, cm	−4.9 (7.8)	−7.1 (7.2)	−1.2 (7.6)	**0.0476**
ΔWaist‐to‐hip ratio	−0.02 (0.05)	−0.02 (0.05)	−0.01 (0.06)	0.602
ΔTotal fat mass, kg	−3.9 (4.8)	−5.3 (5.0)	−1.2 (3.2)	**0.0256**
ΔTotal body fat, %	−2.0 (2.7)	−2.8 (2.9)	−0.7 (1.6)	**0.0389**
ΔAndroid fat, %	−2.9 (4.3)	−3.7 (4.9)	−1.3 (2.7)	0.159
ΔGynoid fat, %	−1.7 (2.5)	−2.2 (2.7)	−0.7 (1.9)	0.147
ΔAbdominal VAT, cm^2^	−25.2 (45.1)	−37.5 (50.1)	−3.0 (22.7)	0.0505
ΔAbdominal SAT, cm^2^	−41.4 (51.7)	−56.8 (51.6)	−13.7 (40.9)	**0.0320**
ΔVAT/SAT ratio	−0.01 (0.09)	−0.02 (0.10)	0.01 (0.04)	0.294
ΔSystolic BP, mmHg	−4.9 (20.5)	−1.5 (19.6)	−10.5 (21.6)	0.259
ΔDiastolic BP, mmHg	−2.3 (12.6)	−1.4 (11.6)	−3.7 (14.5)	0.636
ΔTotal cholesterol, mg/dL	8.2 (14.0)	5.2 (14.8)	13.1 (11.4)^*^	0.141
ΔHDL cholesterol, mg/dL	3.8 (5.4)	4.6 (5.7)^*^	2.5 (4.8)	0.307
ΔLDL cholesterol, mg/dL	7.1 (10.6)	5.0 (11.2)	10.5 (9.1)^*^	0.185
ΔTriglycerides, mg/dL	−13.8 (32.2)	−22.7 (28.3)^*^	0.7 (34.2)	0.0558
ΔHaemoglobin A1c, %	−0.0 (0.6)	−0.0 (0.7)	−0.0 (0.3)	0.996
ΔFasting insulin, mg/dL	−3.7 (12.2)	−3.0 (9.0)	−4.8 (16.7)	0.708
ΔFasting glucose, mg/dL	−1.9 (31.8)	2.4 (38.0)	−9.0 (16.9)	0.356
ΔHOMA‐IR	−1.1 (4.2)	−0.6 (3.1)	−1.9 (5.7)	0.429

*Note*: Table values are mean (SD). Abbreviations: BP, blood pressure; BMI, body mass index; CR, cardiac rehabilitation; CR+WL, cardiac rehabilitation plus a behavioural weight loss intervention; HDL, high‐density lipoprotein; HOMA‐IR, homeostasis model assessment of insulin resistance; LDL, low‐density lipoprotein; SAT, subcutaneous adipose tissue; VAT, visceral adipose tissue.

The bold values indicate statistical significance and are used to highlight the major group differences.

* *p *< 0.05.

### 3.3 Effects of sEV^AT^ on in vitro BBB function

**FIGURE 4:**
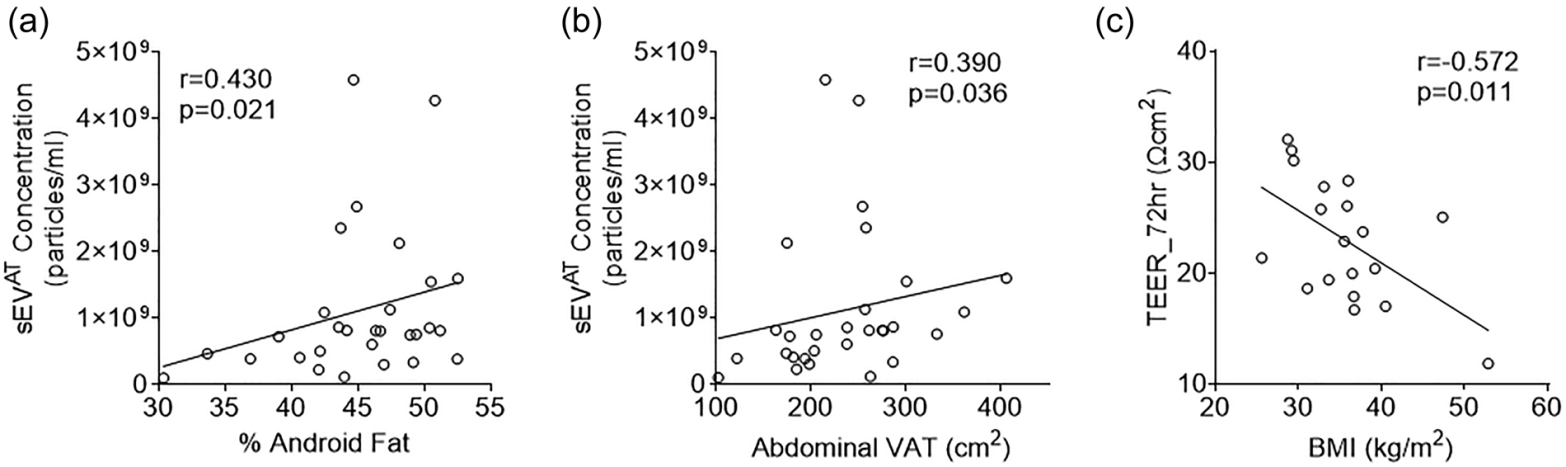
**Effect of sEV^AT^ treatment on brain endothelial cell permeability**. **(a)** Line graph showing the change in TEER over time after treatment of hCMEC/d3 cells with PBS (controls: n=11) and with sEV^AT^ isolated from baseline samples (n=19). Bar graphs representing the mean TEER of controls (n=11), and sEV^AT^‐treated cells from the CR (n=6) and CR+WL (n=13) groups at baseline after incubation for 24 hr **(b)**, 48 hr **(c)** and 72 hr **(d)**. **(e)** Line graph showing the change in TEER over time after treatment of hCMEC/d3 cells with PBS (Controls: n=11) and with sEV^AT^ isolated from follow‐up samples (n=19). **(f)** Bar graphs representing the mean TEER after incubating hCMEC/d3 cells for 72 hr with PBS (controls: n=11) or with sEV^AT^ isolated from CR (n=6) and CR+WL (n=13) groups at follow‐up. Bar graph data are shown as mean ± SD.

Figure 4a shows the time course for changes in TEER following sEV^AT^ treatment at baseline. Our results showed that the average TEER over the 72 h incubation period was generally lower in sEV^AT^‐treated cells versus controls (average effect across all time points, *p *= 0.158), with a significantly lower TEER at 72 h in sEV^AT^‐treated cells compared with controls (23.138 ± 1.209 vs. 28.724 ± 1.613 Ω cm^2^, *p *= 0.012). However, there was no statistically significant difference in TEER responses between the CR and CR+WL groups at baseline (average effect across all time points, *p *= 0.256), with similar TEER values at the 24 (Figure 4b), 48 (Figure 4c) and 72 h (Figure 4d) time points. Similar responses were observed when using samples from the 6 month follow‐up visit (overall *p *= 0.112 for sEV^AT^ vs. control; *p *= 0.001 for sEV^AT^ vs. control at the 72 h time point; *p *= 0.995 for intervention group comparison) (Figure 4e). The 6 month intervention increased TEER at 72 h by 4% in the CR group (pre, 21.8345 ± 1.9527 Ω cm^2^; post, 22.6988 ± 0.6532 Ω cm^2^) but decreased TEER by ∼13% in the CR+WL group (pre, 23.4521 ± 1.6450 Ω cm^2^; post, 20.4459 ± 1.2613 Ω cm^2^) (Figure 4f). Nevertheless, the change in 72 h TEER values from baseline to follow‐up was similar between intervention groups (*p *= 0.199).

### 3.4 Associations with sEV^AT^ characteristics

The sEV^AT^ characteristics were similar in participants with and without obesity (size, *p *= 0.124; particle concentration, *p *= 0.216; protein content, *p *= 0.340) and in those with an abdominal VAT/SAT ratio above versus below the median value of 0.50 (size, *p *= 0.880; particle concentration, *p *= 0.590; protein content, *p *= 0.434). However, cross‐sectional analyses at baseline showed significant positive associations between sEV^AT^ concentrations and adiposity outcomes, including android fat (*r* = 0.430, *p *= 0.021; Figure 5a) and abdominal VAT (*r* = 0.390, *p *= 0.036; Figure 5b). Baseline sEV^AT^ concentration was also positively associated with triglyceride levels (*r* = 0.413, *p *= 0.026). In addition, at baseline, we found a significant association between sEV^AT^ size and high‐density lipoprotein cholesterol (*r* = −0.378, *p *= 0.043). In longitudinal analyses, reductions in sEV^AT^ protein content were significantly associated with reductions in haemoglobin A1c (*r* = 0.368, *p *= 0.050).

### 3.5 Associations with BBB function

At baseline, lower 72 h TEER was significantly associated with higher BMI (*r* = −0.572, *p *= 0.011; Figure 4c), but no other adiposity variables. In longitudinal analyses, decreases in HOMA‐IR were associated with increases in TEER at 72 h (*r* = −0.467, *p *= 0.044). However, there were no statistically significant associations between changes in 72 h TEER values and changes in sEV^AT^ characteristics or adiposity outcomes.

## 4 DISCUSSION

Given the well‐known links between obesity and brain health, particularly in mid‐life, we sought to determine whether sEV^AT^ from adults with overweight and obesity impact BBB function and whether a behavioural weight loss intervention can blunt any potential negative effects. Our study shows, for the first time, that sEV^AT^ increase endothelial permeability (i.e., reduce TEER), as measured using a common in vitro model (Weksler et al., 2013). We also found that sEV^AT^ from individuals with a higher BMI led to greater reductions in TEER compared with those having a lower BMI. Although the behavioural weight loss intervention led to significant reductions in body fat, there were no apparent effects on sEV^AT^ characteristics or changes in the ability of sEV^AT^ to alter TEER. Collectively, our data suggest that sEV^AT^ might have direct effects on the BBB, which might be influenced by the severity of obesity.

sEV and other extracellular vesicles are secreted from multiple cell types and can be found in all biofluids (Colombo et al., 2014). Importantly, these vesicles carry cargo from the cell of origin and reflect the physiological and metabolic state of the cell. Although adipocytes are the primary cells of interest in AT, endothelial cells and macrophages also play key roles in AT function (Inouye et al., 2023) and can release sEV that facilitate interorgan communication. sEV from non‐AT, such as the pancreas, skeletal muscle and liver, are also impacted by obesity and might additionally interact with the brain. Several studies have investigated total sEV in the context of obesity, whereas relatively fewer studies have looked specifically at sEV^AT^. Eguchi et al. (2016) reported that in both mice and humans with obesity, the total sEV concentration in plasma was higher compared with lean control subjects, which was associated with worse glycaemic control. They also found higher plasma sEV^AT^ concentrations in obesity, as estimated by perilipin expression. Similar findings have been observed in two other studies (Camino et al., 2022; Mishra et al., 2023), highlighting the potential utility of sEV^AT^ for studying the effect of obesity on related cardiometabolic diseases. It is also important to note that mesenchymal stem cells in AT, also known as AT‐derived stem cells, can release sEV and have a promising role in regenerative therapies (Papadopoulos et al., 2024). Taken together, these findings suggest that sEV are an important mechanism through which AT can influence both health and disease.

Camino et al. (2022) found that VAT and SAT from patients with obesity secreted higher amounts of sEV^AT^ of larger size compared with lean control subjects. Moreover, these VAT‐ and SAT‐derived sEV differed in their protein composition. These findings are consistent with data from our previous study, where we isolated sEV specifically from VAT and SAT of mice with diet‐induced obesity and lean controls (Mishra et al., 2023). Obese mice had a higher concentration of sEV from VAT compared with lean mice, whereas there was no difference in SAT‐derived sEV concentrations. Furthermore, we reported significantly higher levels of inflammation‐related biomarkers in sEV^AT^ from patients with obesity compared with lean control subjects (Mishra et al., 2023). In the present study, we found similar sEV^AT^ concentrations in obese and non‐obese participants, although the low prevalence of overweight in our population and the lack of a normal‐weight control group are likely to have contributed to the non‐significant findings. However, our results suggest that various measures of central adiposity are positively associated with sEV^AT^ concentrations, consistent with greater sEV^AT^ secretion in adults with a greater burden of obesity. Interestingly, when characterizing participants based on the VAT/SAT ratio, we found no differences in sEV^AT^ concentrations, which differs from our earlier observation in obese mice, where we found greater sEV secretion in VAT versus SAT. This difference might be related to the fact that the specific surface marker used to isolate sEV^AT^ in the present study cannot distinguish between VAT‐ and SAT‐derived sEV. Thus, the translational significance of these findings remains to be elucidated.

Although accumulating data suggest important physiological roles of sEV^AT^ in the context of metabolic diseases (Gao et al., 2017), there is a paucity of data investigating their effects on the brain. A study by Gao et al. (2019) reported that sEV^AT^ from obese mice were internalized by hypothalamic neurons, leading to an increase in body weight and energy intake through modulation of anorexigenic pro‐opiomelanocortin (POMC) expression via the rapamycin (mTOR) signalling pathway. Another study showed that sEV^AT^ can travel to the brain in a membrane protein‐dependent manner, where they are preferentially taken up by neurons and promote synaptic damage and cognitive impairment via microRNA‐9‐3p (Wang et al., 2022). In the present study, we hypothesized that higher sEV^AT^ levels would be correlated with worse BBB function and that body fat would directly impact the effects of sEV^AT^ on BBB function. Our findings suggest that participants with a higher BMI might have worse sEV^AT^‐mediated effects on in vitro BBB permeability. However, the overall effect of sEV^AT^ treatment on TEER was similar in response to the CR and CR+WL interventions. Interestingly, we found that improvements in HOMA‐IR were associated with improvements in sEV^AT^‐induced changes in BBB permeability (as measured by increases in TEER), although the exact mechanisms remain unclear. Given the relatively small sample sizes and wide variability in individual responses, these findings warrant further investigation.

To our knowledge, few studies have investigated sEV^AT^ following weight loss or exercise. In a small sample of adults undergoing bariatric surgery, Witczak et al. (2018) reported that total EV within the 100–200 nm size range was significantly reduced at 6 months postsurgery compared with baseline. The expression levels of EV‐FABP4 (a marker for sEV^AT^) increased significantly over the first month following surgery but then returned to baseline levels by 6 months postsurgery. Moreover, EV‐FABP4 levels were not associated with BMI. In the study by Eguchi et al. (2016), participants with obesity were given a low‐calorie diet for 3 months, during which total EV concentrations were significantly reduced. They also observed a 35% decrease in perilipin A in circulating EV, suggesting a reduction in sEV^AT^ following caloric restriction. Interestingly, greater reductions in total EV concentrations were associated with greater improvements in fasting insulin levels and insulin resistance, but not BMI or total fat mass, possibly because the patients still had a BMI in the obese range. A study by Rigamonti et al. (2020). reported no changes in the total sEV (30–130 nm) or EV‐FABP4 concentrations following an acute bout of moderate‐intensity exercise. The effects of exercise training on sEV^AT^, however, are not known. In our study, the average size and concentration of sEV^AT^ did not change significantly by CR or CR+WL, and the magnitude of changes in these outcomes was not significantly associated with changes in adiposity or cardiometabolic risk factors.

In addition to the size and concentration of sEV^AT^, the protein content is also relevant, because sEVs play an important role in protein transfer and the removal of pathogens (Bellingham et al., 2012). In our earlier study, we found that higher total protein content in circulating neuron‐derived sEV was associated with worse cognitive function, as measured by the MoCA score (Patterson et al., 2018). However, we were unable to replicate this association using sEV^AT^ in the present study. Additionally, we found no significant associations between sEV^AT^ protein content and adiposity and no differences between participants with and without obesity or a higher versus lower VAT/SAT ratio. There were also no significant changes in sEV^AT^ protein content in response to the interventions. We did, however, observe a statistically significant (albeit modest) association with haemoglobin A1c, such that participants with greater reductions in sEV^AT^ protein content tended to have greater reductions in haemoglobin A1c. Although these findings might reflect changes in the transfer of proteins and microRNA that modulate glycaemic control, the relevance of total protein content in sEV^AT^ is unclear. Perhaps what is more important is the specific proteins and other cargo that the sEV^AT^ carry. Indeed, as mentioned above, prior studies by us and others demonstrated that sEV^AT^ from individuals with obesity have a higher expression of several proteins and microRNAs related to immune and inflammatory responses, metabolic processes and other AT‐specific functions (Eguchi et al., 2016; Mishra et al., 2023). Moreover, the contents of sEV^AT^ are modifiable, and both diet‐ and surgery‐induced weight loss lead to notable improvements in pathways related to fat storage, insulin signalling and metabolism of branched‐chain amino acids, which are correlated with improvements in glycaemic control (Eguchi et al., 2016; Hubal et al., 2017; Witczak et al., 2018). Given that we did not measure specific sEV^AT^ proteins in this study, we can only speculate how changes in proteins and other molecules involved in different pathways (e.g., glycaemic control, inflammation, lipid metabolism) might change in relationship to relevant clinical outcomes (e.g., insulin resistance).

There are several limitations that should be considered. The study population was small and included mostly White men, which limits generalizability. Given the well‐known differences in body composition and body fat distribution in males and females and the reported sex differences in sEV characteristics (Noren Hooten et al., 2022), it is possible that the effects of sEV^AT^ on BBB might be sex dependent. As mentioned previously, only participants with overweight or obesity were included in the present study, and there was no lean control group. This limited our ability to detect associations between the sEV^AT^ and adiposity outcomes. Moreover, with the present study design, including the sole reliance on PBS as the control condition for the TEER experiments, we cannot confirm the effects of sEV^AT^ on BBB function in healthy versus diseased states, which is important given the potential protective effect of sEV.

Another limitation is the sole reliance on FABP4 to isolate sEV^AT^ from plasma. This protein has been widely used as a marker for differentiated adipocytes (Shan et al., 2013), and although it is most abundant in AT, it is also expressed in other cell types, including endothelial cells and macrophages (Inouye et al., 2023). AT biopsies would be an ideal approach to obtain sEV^AT^; however, direct sampling of AT is invasive, and some key AT depots (i.e., visceral AT) can be difficult to access. Plasma levels of circulating sEV^AT^ can therefore serve as a less invasive ‘liquid biopsy’ to provide useful information about the physiological status of AT. Given our recent study validating the use of multiple surface markers (including FABP4) to isolate sEV^AT^ (Mishra et al., 2023), the approach used in the present study might have resulted in a significantly higher but less specific yield of plasma sEV^AT^.

It is also important to note that BBB function was assessed using the hCMEC/d3 cell line, a single cell model of the BBB. Although it is useful for testing endothelial permeability, it is not a complete representation of the BBB, because besides endothelial cells, the BBB also contains pericytes and astrocytes (Qi et al., 2023). In addition, we measured only TEER, which reflects the physical barrier properties of the BBB, but not properties related to tight junctions, metabolic enzymes and transport proteins that reflect other BBB functions. Nevertheless, our study provides the first evidence that sEV^AT^ from human plasma can alter the permeability of the BBB using an in vitro assay.

## 5 CONCLUSION

In summary, our study suggests that sEV^AT^ from adults with overweight and obesity might adversely affect BBB function, as measured by TEER, which might be driven by excessive amounts of dysfunctional AT. Despite the favourable effects of CR+WL on body composition and body fat distribution, there were no notable changes in sEV^AT^ or their ability to modulate in vitro BBB function with exercise or weight loss. Future studies should focus on replicating these experiments in larger, more diverse populations using a more physiologically relevant model of BBB function (e.g., three‐dimensional brain organoids) and more robust controls, such as sEV^AT^ from lean individuals and sEV from non‐AT, to confirm the biological effects of sEV^AT^ in the context of obesity. Using depot‐specific markers of sEV^AT^ will further advance the field by helping to determine the role of VAT versus SAT in interorgan crosstalk with the brain. Investigating additional metrics of endothelial permeability and the expression of adhesion and transport proteins will be necessary to characterize BBB function better. Lastly, omics‐based approaches will be crucial in identifying the components of sEV^AT^ that are modifiable with intervention and drive changes in BBB function.

## AUTHOR CONTRIBUTIONS

Study conception and design: Shalini Mishra, Fang‐Chi Hsu, Gagan Deep and Tina E. Brinkley. Acquisition, analysis or interpretation of the data: All authors. Drafting and critical revision of the manuscript: All authors. All authors approved the final version of the manuscript and agree to be accountable for all aspects of the work in ensuring that questions related to the accuracy or integrity of any part of the work are appropriately investigated and resolved. All authors meet the criteria for authorship, and all individuals who qualify for authorship have been included in the author list.

## CONFLICT OF INTEREST

G.D. is the founder of LiBiCo LLC, which has no influence or contribution to the work presented in this manuscript. All other authors declare no conflict of interest.

## Data Availability

Data will be made available to investigators upon reasonable request and completion of a data‐sharing agreement, which will require that the data be kept secure and used only for research purposes.
